# Long‐term assessment of adverse cardiovascular events in men receiving intermittent androgen deprivation therapy following radical prostatectomy

**DOI:** 10.1002/bco2.70127

**Published:** 2025-12-12

**Authors:** Joshua Tran, Yeagyeong Hwang, Mai Xuan Nguyen, Gillian Mendoza, Erica Huang, Linda Huynh, Rafael Gevorkyan, Catherine Fung, Robert Wilson, Sheldon Greenfield, Thomas Ahlering

**Affiliations:** ^1^ Department of Urology University of California Irvine CA USA; ^2^ University of Massachusetts Worcester MA USA; ^3^ MD/PhD Scholars Program University of Nebraska Medical Center Omaha NE USA; ^4^ Health Policy Research Institute University of California Irvine CA USA

**Keywords:** adverse cardiovascular events, androgen deprivation therapy, biochemical recurrence, cardiovascular risk, radical prostatectomy

## Abstract

**Objectives:**

To assess the relationship of intermittent adverse cardiovascular events (ACE) in men undergoing androgen deprivation therapy (ADT) post radical prostatectomy (RP), since ACE are severe complications associated with ADT following a biochemical recurrence (BCR) post‐RP for the treatment of prostate cancer (PC).

**Patients and Methods:**

Retrospective review of prospectively collected data of patients who underwent robot‐assisted radical prostatectomy (RARP) with a BCR (n = 407). A total of 308 men with adequate follow‐up data included for analysis. A total of 189/308 men in the “treatment group” (TG) were managed with ADT. The comparator group consisted of 119/308 men with no treatment (NT). Regression and Kaplan Meier (KM) analyses were performed to assess predictors of ACE.

**Results:**

At baseline, patients in the treatment group had higher risk characteristics for PC (preoperative PSA, pathological stage and Gleason grade). Univariate analysis of ACE showed significant predators were age, Charlson comorbidity index (CCI), body mass index (BMI), treatment status and smoking status. In multivariate analysis, treatment status was trending towards significance (p = 0.10) with CCI (p < 0.001) and BMI (p = 0.003) being significant predictors of ACE. In 15‐year KM, we observed a significant increase in ACEs (TG 54.4% and NT 41.8%, p = 0.02). Limitations include retrospective design and limited analysis of NT, TG or ADT effects on cardiovascular mortality.

**Conclusion:**

ADT, in our experience, is associated with an increased risk of ACE. We also noted the importance of CCI and BMI as a prognosticating tool for ACE.

## 1 INTRODUCTION

Prostate cancer (PC) is the most commonly diagnosed non‐cutaneous cancer in men, with about 270,000 new cases diagnosed in the United States, with an estimated 34,500 deaths in 2022.^1^ The most common surgical treatment for PC is a radical prostatectomy (RP). Patients are considered to have biochemical recurrence (BCR) following two elevated prostate‐specific antigen (PSA; > 0.2 ng/ml) blood tests post RP. Patients presenting with a BCR, occurring in about 20–40% of patients, are recommended to receive androgen deprivation therapy (ADT) alone or radiation therapy (RT) with or without ADT. These treatments are utilized to help prevent mortality from PC but can come with harmful side effects.^2^ An increase in cardiovascular disease (CVD) has been associated with use of ADT; however, these complications have been highly debated with conflicting results in the literature.

Several studies have assessed the use of hormonal therapy and the risks of cardiovascular mortality (CM). One of the most well‐known publications on this topic is a science advisory from the American Heart Association, American Cancer Society and American Urological Association that was published in 2010.^3^ Despite the growing literature on a relationship between PC patients on ADT and increased risk of CVD, the consensus states that “there may be a relationship” but does not believe that patients should be referred to additional specialists or perform any additional tests to ensure safety.

Nguyen et al. in 2011, reported a meta‐analysis of 11 randomized control trials (RCTs) that found no significant difference in CM between patients receiving ADT vs control.^4^ RCTs have continuously reported ADT use does not increase CM in patients.^5^, ^6^, ^7^, ^8^ While RCTs are considered one of the highest levels of evidence, observational studies report differing results.^9^, ^10^, ^11^, ^12^, ^13^, ^14^ In 2015, Bosco et al. published a meta‐analysis on observational studies with eight studies reporting the use of ADT and a nonfatal or fatal CVD outcome. They found that observational studies consistently show a positive association between ADT use and risk of CVD.^15^

Contemporary literature cannot agree on the association of ADT and CM. EAU guidelines^16^ state level I evidence conclude conflicting results and can only give advice on “non‐specific measures such as loss of weight, increased exercise minimizing alcohol intake, and smoking cessation.” Our study is the first to compare the relationship of adverse cardiovascular events (ACE) in men (post‐RP) suffering a BCR who underwent treatment versus a BCR who did not have treatment.

## 2 METHODS

Retrospective review of prospectively collected data from patients who underwent robot‐assisted RP performed by a single surgeon between June 2002 and September 2019 (N = 1895) at a tertiary referral centre. All study data were prospectively collected under an approved institutional review board at the University of California, Irvine (#1998–84). Data were obtained from electronic medical records, pathology reports and standardized postoperative follow‐up assessments, including laboratory tests and imaging. All data collection was conducted in compliance with the Health Insurance Portability and Accountability Act and federal guidelines for informed consent were followed.

**FIGURE 1:**
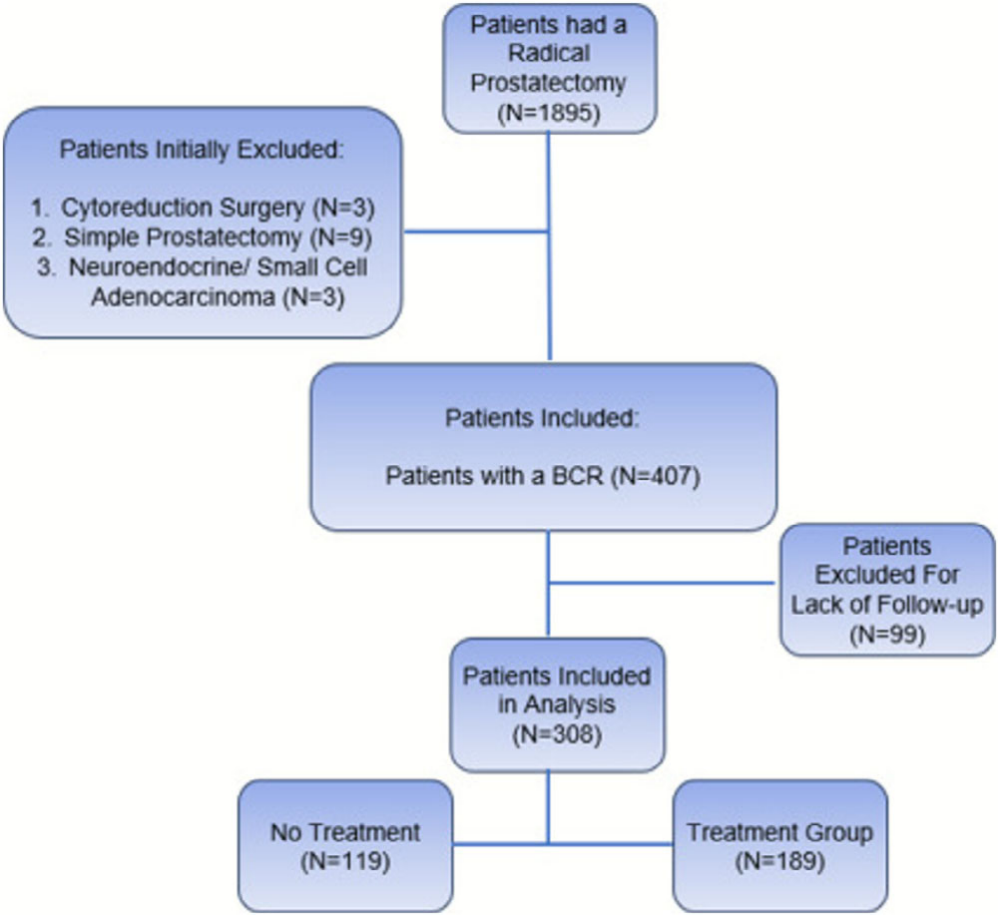
Inclusion and Exclusion Criteria for patient population.

Patients were initially screened and excluded if they underwent simple prostatectomy (N = 9), cytoreductive prostatectomy (N = 3), had neuroendocrine or small cell carcinoma (N = 3). A total of 407 patients had a BCR post‐RP and were included in the study (Figure 1). Follow‐up data were collected until March 29, 2021, with 99 patients excluded due to lack of follow‐up. The final cohort consisted of 308 patients that either received ADT treatment (Lupron 45mg for 6‐month administration) (TG; Treatment Group, N = 189) or did not receive treatment (NT; No Treatment group, N = 119) (Table 1). Patients underwent four cycles of ADT over a 2‐year period, after which treatment was discontinued. ADT was subsequently reinitiated when the PSA level rose to 4.0 ng/ml. Patients on intermittent ADT had an average of 1.76 cycles with an average course of 2 years on ADT per cycle. The average length of holiday was 1.5 years.

**TABLE 1:** Descriptive statistics of No Treatment (NT) and Treatment (TG) groups.

Treatment	NT	TG	Total	
	**Count (%)**	**Count (%)**	**Count (%)**	
N, all patients	119 (38.6%)	189 (61.4%)	308 (100%)	
	**Mean (SD)**	**Mean (SD)**	**Mean (SD)**	**P value**
Age, years	63.3 (7.34)	64.2 (7.25)	63.8 (7.29)	0.2919
Adj Pre‐PSA, ng/mL	8.19 (5.30)	12.9 (17.9)	11.1 (14.6)	**0.0056**
Body mass index	27.2 (3.80)	27.2 (3.91)	27.2 (3.86)	1.0000
Charlson Comorbidity Index	4.15 (1.04)	4.38 (1.19)	4.29 (1.14)	0.0845
Follow‐up, years	7.37 (39.8)	7.69 (4.42)	7.57 (4.25)	0.5210
	**Count (%)**	**Count (%)**	**Count (%)**	**P value**
p‐stage				**< 0.001**
pT2	59 (49.6%)	46 (24.3%)	105 (34.1%)	
pT3/T4	60 (50.4%)	143 (75.7%)	203 (65.9%)	
Gleason Grade Group				**< 0.001**
1	14 (11.8%)	1 (0.5%)	15 (4.9%)	
2	42 (35.3%)	35 (18.5%)	77 (25.0%)	
3	41 (34.5%)	57 (30.2%)	98 (31.8%)	
4	12 (10.1%)	13 (6.9%)	25 (8.1%)	
5	10 (8.4%)	83 (43.9%)	93 (30.2%)	
Patients with ACE				0.0625
No Events	97 (81.5%)	135 (71.4%)	232 (75.3%)	
At Least 1 ACE	22 (18.5%)	54 (28.6%)	76 (24.7%)	
Smoking Status				0.8613
Non‐Smoker	78 (65.5%)	127 (67.2%)	205 (66.6%)	
Previous Smoker	41 (34.5%)	62 (32.8%)	103 (33.4%)	

The primary outcome was defined as the presence or absence of at least one ACE. An “event” date was noted only if an ACE occurred post‐RP in the NT group and post‐ADT in the treatment group. ACE was measured according to Zhang et al. based on the FDA Adverse Event Reporting System,^17^ which includes coronary artery disease (CAD), arrhythmia, myocardial infarction (MI), ischemic stroke, congestive heart failure (CHF), deep venous thrombosis (DVT), pulmonary embolism (PE) and peripheral vascular disease (PVD). While some events are included in the Charlson Comorbidity Index (CCI),^18^ these events were not counted unless an additional event was noted post‐RP.

T‐tests were utilized for continuous variables, and chi‐squared analysis to evaluate categorical variables. A backward stepwise regression model was utilized to find independent predictors of ACE. In multivariate modelling, independent variables were initially chosen a priori. Ad hoc logistic regression analysis explored the effects of smoking status as an independent variable.

A 15‐year cardiac event survival assessment was performed with Kaplan–Meier analysis and stratified between the TG and NT groups. Statistical significance was defined as a p‐value <0.05 for all statistical testing. All statistical tests and figures were conducted and produced in the R statistical package (R Foundation for Statistical Computing, Vienna, Austria).

## 3 RESULTS

Both groups had similar age (NT: 63.3 yrs vs TG: 64.2 yrs) and follow‐up time (NT: 7.37 yrs vs TG: 7.69 yrs). Treatment patients had a higher PC risk profile distribution with respect to preoperative PSA (Pre‐PSA), Gleason Grade group (GGG) and pathologic stage (p‐stage) (Table 1). No other variables reached statistical significance.

Univariate regression analysis found age, CCI, BMI, treatment status and smoking status were statistically significant predictors of ACE. Multivariate analysis showed CCI sum and BMI were significant predictors of ACE. It is important to note that although treatment status was not statistically significant in the final model, the variable was trending towards significance (p = 0.10) (Table 2).

**TABLE 2:** Univariate and multivariate logistic regression analysis for ACE.

	Univariate		Multivariate	
Variable	OR (95% CI)	P‐Value	OR (95% CI)	P‐Value
Age	1.08 (1.04, 1.12)	**<0.001**	‐‐	‐‐
Adjusted Gleason Grade Group			**‐‐**	**‐‐**
1	‐‐	‐‐		
2	1.22 (0.34, 5.78)	0.78		
3	1.16 (0.33, 5.41)	0.83		
4	2.25 (0.54, 11.8)	0.29		
5	1.39 (0.40, 6.48)	0.63		
Charlson Comorbidity Index	1.67 (1.32, 2.14)	**<0.001**	1.7 (1.31, 2.23)	**<0.001**
BMI	1.1 (1.03, 1.18)	**0.005**	1.12 (1.04, 1.20)	**0.003**
Treatment				
NT	‐‐	‐‐	‐‐	‐‐
TG	1.76 (1.02, 3.14)	**0.047**	1.65 (0.91, 3.07)	**0.1**
Adjusted Pre‐PSA	1 (0.97, 1.01)	0.7		
Adjusted Pathologic Stage			‐‐	‐‐
pT2	‐‐	‐‐		
pT3	1.49 (0.85, 2.67)			
Smoking Status*				
Non‐Smoker	‐‐	‐‐		
Previous Smoker	2.06 (1.21, 3.51)	**0.008**		

OR = Odds Ratio, CI = Confidence Interval, PSA = Prostate Specific Antigen.

* Not assessed in initial Multivariate analysis.

Ad hoc analysis of smoking status' effect on ACE is outlined in Table 3. The initial model included all independent predictors of ACE along with smoking status. An interim model was included to outline a significant trend between age and CCI sum. While the final model had CCI sum fall out, it was close to statistical significance and should remain an important factor to consider. The final model for this analysis found age and BMI as significant predictors of ACE with treatment status trending (p = 0.074), but no longer statistically significant.

**TABLE 3:** Multivariate logistic regression model of ACE, adjusting for covariates that affect aggressiveness of disease and cardiovascular morbidity.

Model	Variables	OR	95% CI	p‐value
Initial Model	Age	1.06	1.00, 1.12	**0.043**
	Adjusted Gleason Grade Group			
	1	**‐‐**	**‐‐**	
	2	0.73	0.17, 3.88	0.68
	3	0.44	0.10, 2.39	0.298
	4	1.06	0.20, 6.56	0.945
	5	0.43	0.09, 2.47	0.306
	Adjusted Pre‐PSA	0.99	0.96, 1.02	0.64
	Adjusted Pathologic Stage			
	pT2	‐‐	‐‐	
	pT3	1.11	0.56, 2.22	0.771
	Charlson Comorbidity Index	1.37	0.97, 1.97	0.07
	Treatment			
	NT	‐‐	‐‐	
	TG	2.14	1.09, 4.34	**0.03**
	BMI	1.14	1.06, 1.23	**<0.001**
	Smoking Status			
	Non‐Smoker	‐‐	‐‐	
	Previous Smoker	1.46	0.78, 2.69	0.228
Interim Model	Age	1.05	1.00, 1.11	0.061
	Charlson Comorbidity Index	1.36	0.97, 1.91	0.073
	Treatment			
	NT	‐‐	‐‐	
	TG	1.7	0.93, 3.18	0.089
	BMI	1.13	1.05, 1.22	**0.001**
	Smoking Status			
	Non‐Smoker	‐‐	‐‐	
	Previous Smoker	1.59	0.87, 2.87	0.127
Final Model	Age	1.08	1.04, 1.13	**<0.001**
	Treatment			
	NT	‐‐	‐‐	
	TG	1.73	0.96, 3.22	0.074
	BMI	1.13	1.05, 1.22	**0.001**
	Smoking Status			
	Non‐Smoker	‐‐	‐‐	
	Previous Smoker	1.58	0.87, 2.83	0.127

OR = Odds Ratio, CI = Confidence Interval.

**FIGURE 2:**
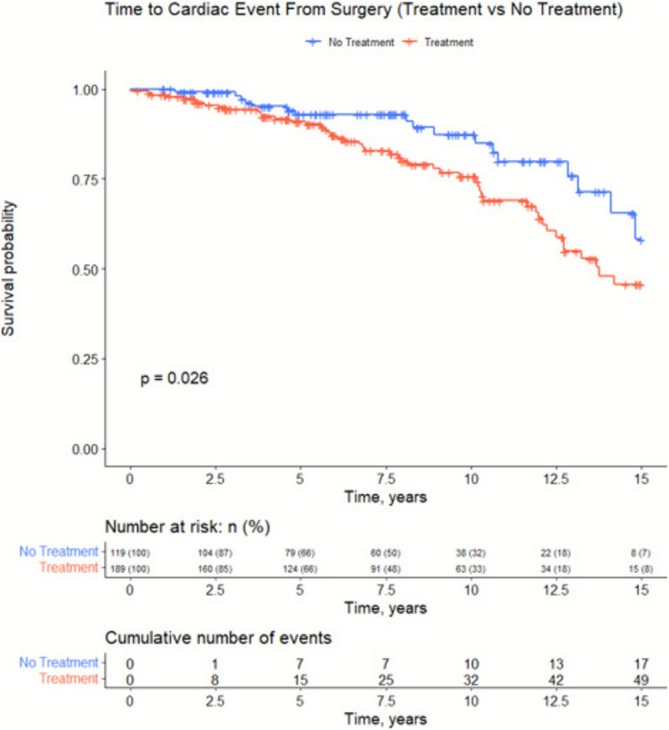
15‐year Kaplan–Meier analysis of ACE survival between no treatment (NT; n = 119) and treatment group (TG; n = 189). Time to ACE calculated from time of RP.

A 15‐year Kaplan–Meier survival curve found a statistically significant difference (p = 0.026) in ACE between the two groups (Figure 2). Patients in the TG had more ACEs when compared to NT patients with the difference increasing the longer out from surgery.

## 4 DISCUSSION

Initial comparison of ACE between NT and TG showed no difference between the groups, with TG patients at a higher risk and trending towards significance (Table 1). This result was reinforced with univariate analysis showing treatment status to be a statistically significant predictor of ACE (Table 2). Although treatment status was no longer significant in multivariate analysis, it was often trending towards statistical significance (Table 3). Highlighting that treatment status may not be an independent predictor of ACE but remains important for physicians to consider when managing a patient's care. Similarly, smoking status, while not statistically significant, is shown to be an important factor for the risk of ACE. Our results may have been confounded by the unknown cessation history of our patients. History of smoking has been shown to be a contributing factor for ACEs and cardiovascular health. This is supported by the literature and recommendation of the American Heart Association.^19^, ^20^, ^21^

In our study, BMI and CCI were found to be significant predictors of ACE. While these are factors that are not typically managed by a urologist, it remains crucial to evaluate before prescribing ADT or hormonal therapy. In 2022, A consensus panel of 117 international prostate cancer experts,^22^ could not agree on which routine evaluation of cardiovascular health should be performed prior to initiation of hormonal therapy. However, they did provide a general recommendation outlining how vital it was for strong communication between prostate cancer specialists and general practitioners in managing side‐effects and long‐term care of the patient.

A strength of our study was the ability to limit the effects of disease burden. While the treatment group had more patients with higher‐risk disease, both groups had BCR. None of our regression models suggests an association between disease aggressiveness (GGG, Pre‐PSA, p‐stage) and ACE. Despite the differences in these variables, our analysis shows that the differences in patient's demographics are not a contributing factor for ACEs. A possible connection can be argued that treatment status may be a mediator between disease aggressiveness and ACE. This is outside the scope of this study but poses a fascinating direction for future studies.

Results from the Kaplan–Meier analysis may explain why there is such a stark difference in results between RCTs^4^ and observational studies.^23^ RCTs, especially those funded by grants, are not often designed to follow patients beyond several years. Our analysis shows that patients are more likely to experience an ACE beyond 10 years post‐op, with risks increasing as time goes on. While treatment status was often no longer significant in our analyses, Figure 2 concludes that there may be a time‐varying component to ACEs that is not considered in regression analysis. The Kaplan–Meier curve displays a growing difference between NT and TG patients' ACE survival as time passes. Emphasizing this result is paramount as patients often live beyond 10 years post‐RP and do not die from PC.^24^ Patients that are expected to live longer than 10 years post‐RP are at a significantly higher risk of experiencing an ACE if they have received treatment.

The trial by Bill‐Axelson et al., found PC specific mortality was only 19.6% at 23 years.^25^ Their findings support the idea that clinicians should carefully weigh recurrence and long‐term prostate‐specific mortality with adverse effects of treatment such as ADT. A previous study by Huang et al. shows that a subset of patients can be observed without the need for treatment.^26^ If patients fall into this group and are at a higher risk of cardiovascular morbidity, it may be more advantageous for that subgroup of patients to not receive treatment. In a separate paper, the specific methodology of observation is described that can safely monitor and manage these patients.^27^

### 4.1 Limitations

This study is unable to analyse the effect of NT and TG on cardiovascular‐related mortality. Our cohort experienced a relatively low number of deaths (14.6%) and an even lower number of cardiovascular‐related mortality (2.6%). It has been noted that between 75% and 83% of patients are managed with continuous ADT, while our patients were managed with intermittent ADT.^28^ This study was also conducted at a single institution and largely reflects the experience of one high‐volume surgeon, which may limit generalizability. Another limitation is the lack of standardized treatment regime across institutions and practice for ADT duration. Due to this, we were unable to compare the effects of intermittent and continuous ADT and their effects on ACEs. This should be explored in future studies.

While our work has outlined the predictors of ACE, many aspects of our study can be utilized as the basis for future studies. Possible future studies may wish to look at different thresholds for BMI, CCI and age. Analysis of thresholds may lend to advising physicians of what may be considered a “high” risk patient. Grouping these patients into different groups may also change the outcome of similar analyses performed in this study.

## 5 CONCLUSION

The present study establishes that there is an association between treatment for BCR and subsequent cardiovascular morbidity (as measured by ACE). Significant predictors of ACE are established, illustrating the importance of BMI and CCI sum. Treatment status remains an important risk factor, as it continues to be trending towards statistical significance in most of our models. Utilizing these predictors, we can stratify patients into different risk groups for ACE based on information collected at the time of RP. These patients would need to be carefully examined to determine if the risks of disease progression outweigh the risks of cardiovascular morbidity. Future work is required to rigorously inform potential modifications to guidelines for prognosticating the effect of treatment on patients to help inform physicians of the possible risks of post‐treatment ACE.

## AUTHOR CONTRIBUTIONS

J.T. conceived the study and planned the analyses. J.T., Y.H., M.X.N. and R.G. collected and curated the data. J.T., Y.H., E.H. and L.H. performed the formal statistical analyses. J.T., Y.H., M.X.N., G.M., R.G. and C.F. contributed to the investigation and data interpretation. M.X.N., G.M. and R.G. prepared the visualizations. C.F. contributed to the study methodology. R.W., S.G. and T.A. supervised the project and provided guidance throughout. J.T. took the lead in drafting the manuscript. Y.H., R.W., S.G. and T.A. contributed to reviewing and editing the manuscript. All authors reviewed the manuscript.

## CONFLICT OF INTEREST STATEMENT

The authors have no conflicts of interest to disclose.

## Data Availability

The datasets generated during and/or analysed during the current study are available from the corresponding author on reasonable request.
